# Clinical particularities in an atypical case of retinitis pigmentosa

**Published:** 2016

**Authors:** Stanca T Horia, Tabacaru Bogdana, Suvac Elena

**Affiliations:** * “Prof. Dr. Agrippa Ionescu” Clinical Emergency Hospital, Bucharest, Romania; ** “Metropolitan Hospital” Bucharest, Romania; *** “Carol Davila” University of Medicine and Pharmacy, Bucharest, Romania

**Keywords:** Cystoid Macular Oedema, Optic Disc Drusen, Retinitis Pigmentosa, Retinitis Punctata Albescens

## Abstract

We present a case of Retinitis Pigmentosa with atypical aspect of fundus (Punctata Albescens), associated with Cystoid Macular Oedema and Optic Disc Drusen.

## Introduction

Retinitis Punctata Albescens is a progressive rod-cone dystrophy, with autosomal recessive transmission [**1**], that can be regarded as one of the subtypes [**2**] atypical variant [**3**,**4**] or incomplete forms of Retinitis Pigmentosa [**3**]. The symptomatology and paraclinical investigations in Retinitis Punctata Albescens are similar to those in classical Retinitis Pigmentosa. The fundus aspect in Retinitis Punctata Albescens presents multiple discrete white spots, especially scattered toward the equator [**3**,**4**].

## Material and methods – Case report

A 34-year-old Caucasian woman first presented in our clinic in 2012 complaining of nyctalopia and progressive visual field loss, symptoms with a relatively sudden onset one year before, after the second child birth. In the past, she presented no consanguinity, no relevant family history and no systemic diseases, but several allergic reactions.

At presentation, her best-corrected visual acuity was 20/ 20 on both eyes, with a small spherocylindrical myopic correction. By the applanation of tonometry, the intraocular pressure was 11 mmHg in the right eye and 10 mmHg in the left eye.

The findings of the anterior pole on the external examination and slit-lamp examination were within normal limits.

**Fig. 1:**
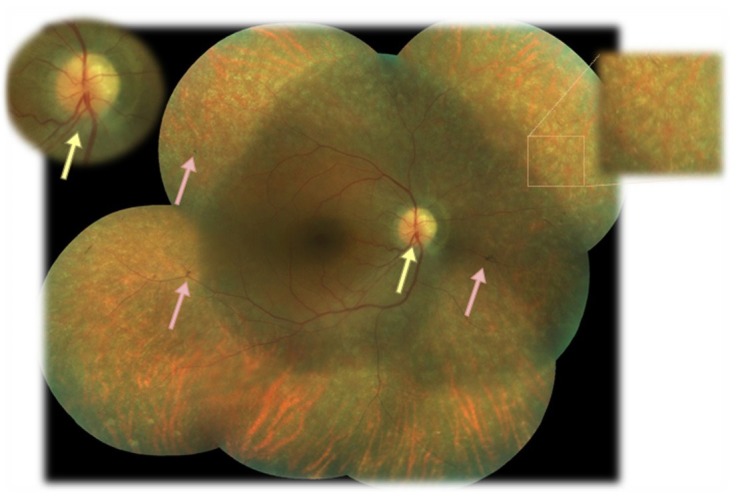
Fundus in the right eye of the patient

**Fig. 2:**
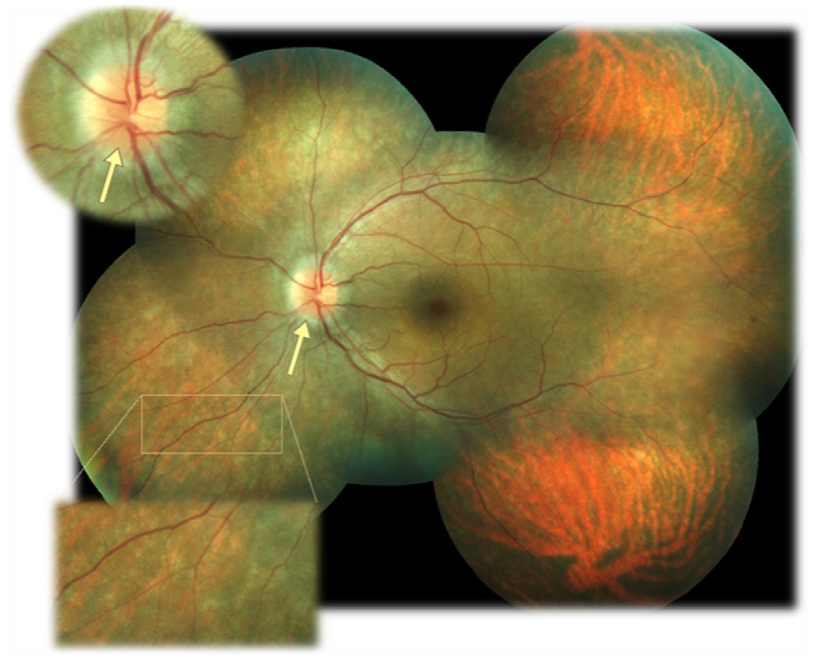
Fundus in the left eye of the patient

The fundus of each eye was examined after a pharmaceutical mydriasis with 0.5% tropicamide and 10% phenylephrine hydrochloride ophthalmic solutions (**Fig. 1**,**2**). The optic nerve disc was imprecisely delimited, had a swollen appearance, the retinal vessels exited centrally, there was no cupping (yellow arrows), that aspect being highly suggestive of drusen of the disc. The retinal arteries were narrowed. Macula appeared to be atrophic, with intraretinal cystic areas. Extramacular, there were multiple white and yellow spots scattered throughout the retina.

 Only the right eye presented three spicule pigment depositions with “bone corpuscle” aspect (pink arrows).

**Fig. 3:**
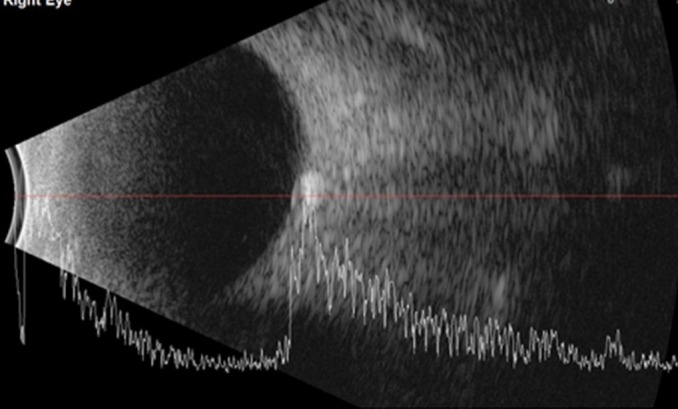
B-scan ultrasonography in the right eye

**Fig. 4:**
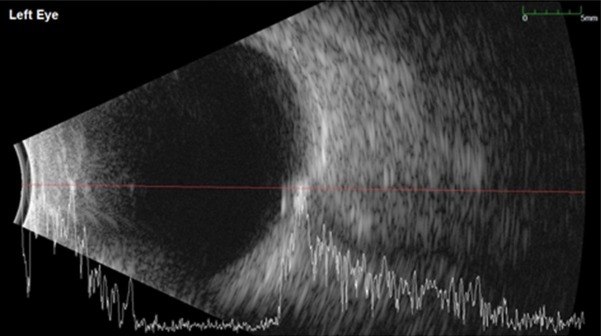
B-scan ultrasonography in the left eye

The B-scan ultrasonography showed an ovoid lesion at the junction of the retina and optic nerve head with a high acoustic reflectivity, which confirmed the optic nerve drusen (**Fig. 3**,**4**).

**Fig. 5:**
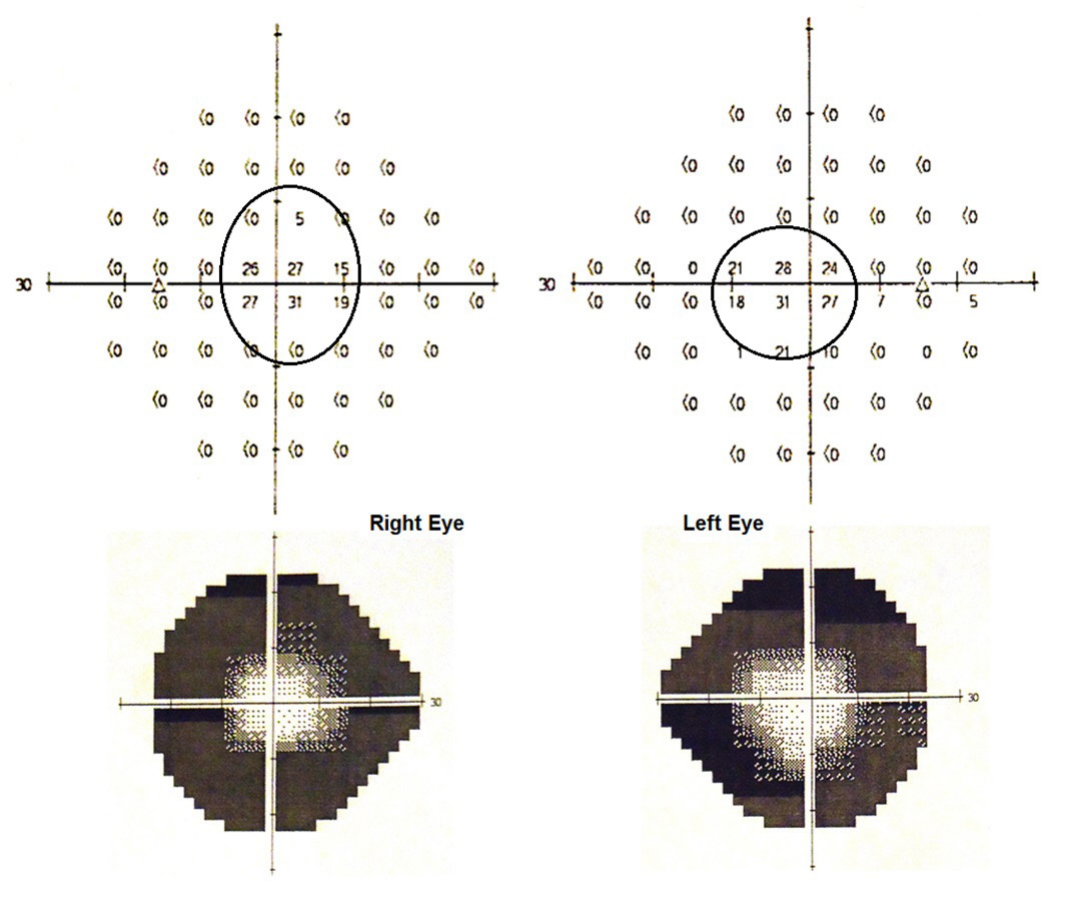
Threshold values maps and grayscale maps from Humphrey visual field of both eyes

Perimetry was assessed by a Humphrey visual field analyzer, central 24-2 threshold program, with a size III white stimulus. Reliability indices were very good in the visual fields of both eyes. Perimetry demonstrated a tunnel vision and absolute scotoma in all quadrants outside the limit of the central 10 degrees, in both eyes (**Fig. 5**).

**Fig. 6:**
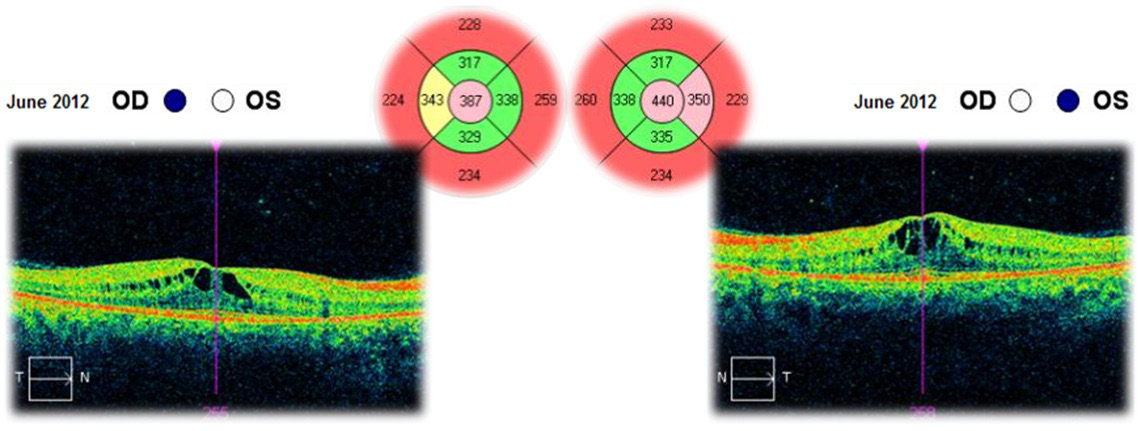
Optical coherence tomography showing an increased retinal thickness due to cystoid macular oedema in both eyes

**Fig. 7:**
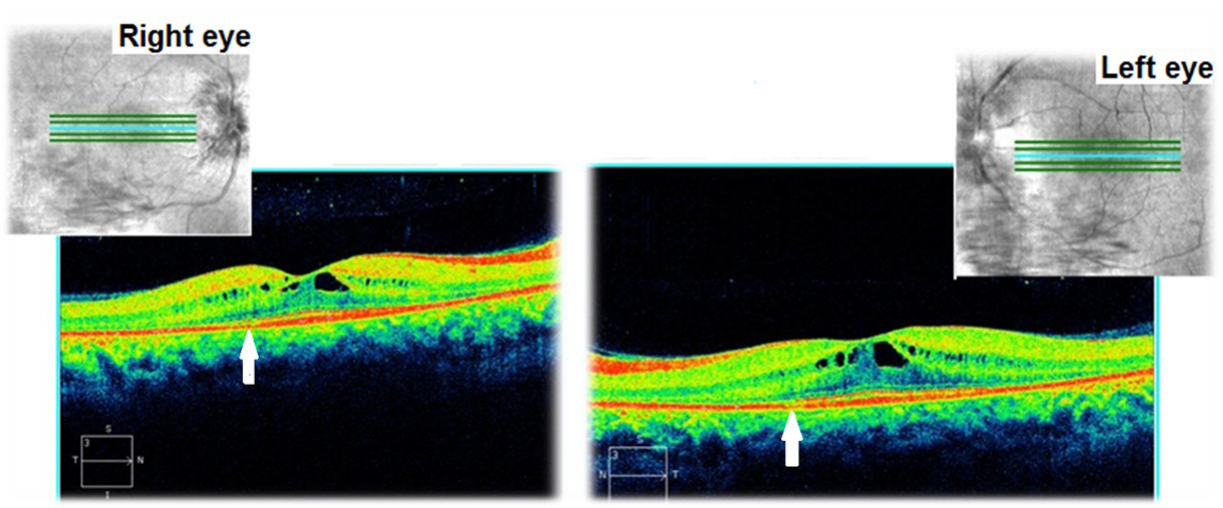
High-resolution OCT images in both eyes

The optical coherence tomography (OCT) of the macula revealed an increased retinal thickness in both eyes, due to multiple areas of low reflec- tivity corresponding to intraretinal cysts and fluid accumulation (**Fig. 6**). The structure of the photo- receptors layer was analyzed on high-resolution OCT scans. It had a normal structure in the foveal region, but in the parafoveal one, discontinuity to the outer segments of the photoreceptors (white arrows) was found. Cystoid spaces were also con- firmed by high-resolution OCT images (**Fig. 7**).

Because of the allergic conditions of the pa- tient, a fluorescein angiography could not be per- formed.

**Fig. 8:**
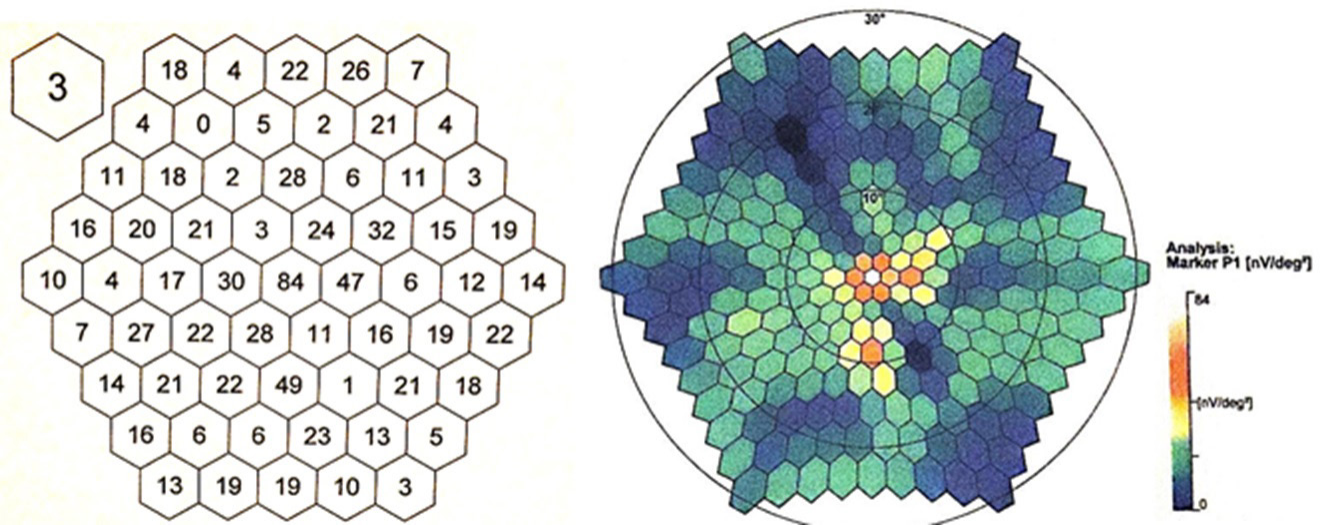
A two-dimensional presentation of P1 amplitudes on mfERG in the right eye

**Fig. 9:**
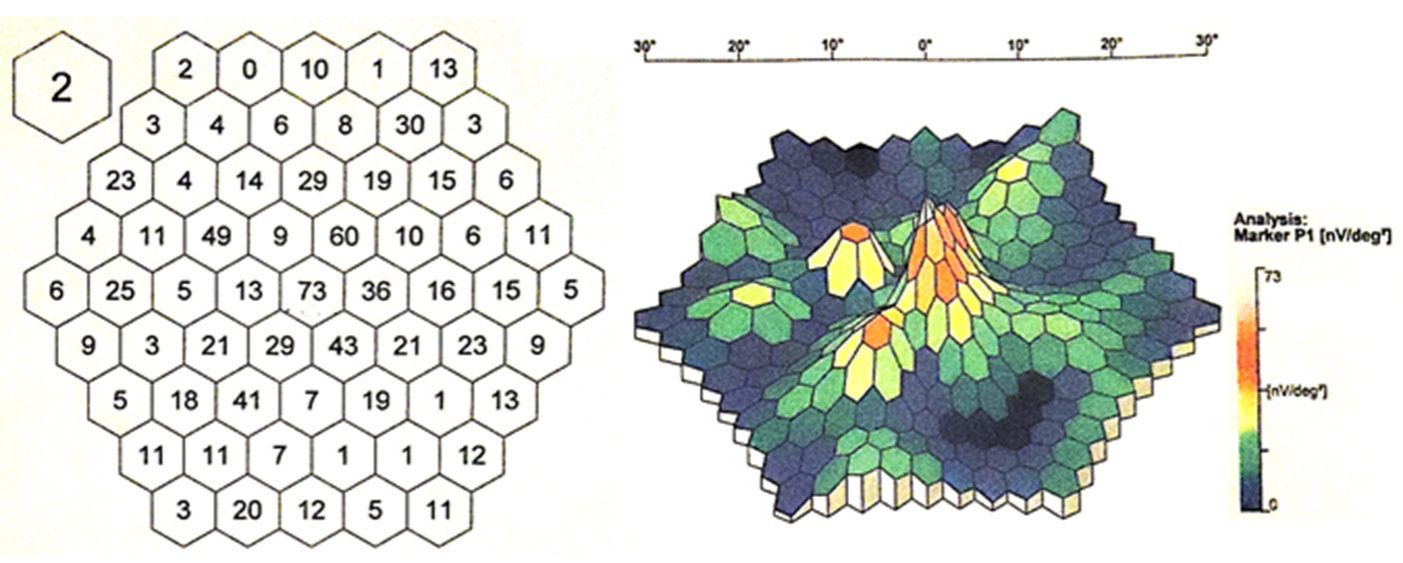
A two-dimensional presentation of P1 amplitudes on mfERG in the left eye

The multifocal electroretinogram (mfERG) using the 61-hexagon stimulus showed signif- icant reductions in response to the amplitudes in the extramacular areas and a higher but also inappropriately lower response amplitude at the fovea (**Fig. 8**,**9**).

Based on the fundus aspect and paraclinical investigations, the diagnosis of Retinitis Punctata Albescens (variant of Retinitis Pigmentosa), Cystoid Macular Oedema, and Optic Disc Drusen was established in both eyes.

**Fig. 10:**
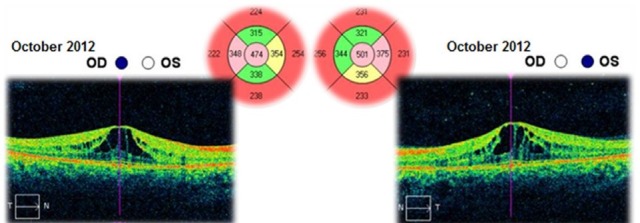
Macular OCT in October 2012 – Cystoid macular oedema – Central retinal thickness is higher than in the previous scan (June 2012) – with 87μm in the right eye and 61μm in the left eye

The patient was followed-up for 3 years. This time she had received a treatment with systemic carbonic anhydrase inhibitors (acetazolamide) for several times and antioxidant supplementation. The best corrected visual acuities in both eyes varied from 20/ 20 (in June 2012) to 20/ 25 (in August 2015). The cystoid macular oedema never regressed completely and the macular thickness varied between 365 – 537μm in the right eye and between 329 – 501μm in the left eye (**Fig. 10**-**Fig. 15**). In the last OCT scan (August 2015), the photoreceptors layer in the fovea appeared to be interrupted and, in the parafoveal regions, the disappearance of photoreceptors (**Fig. 15**) was noticed.

**Fig. 11:**
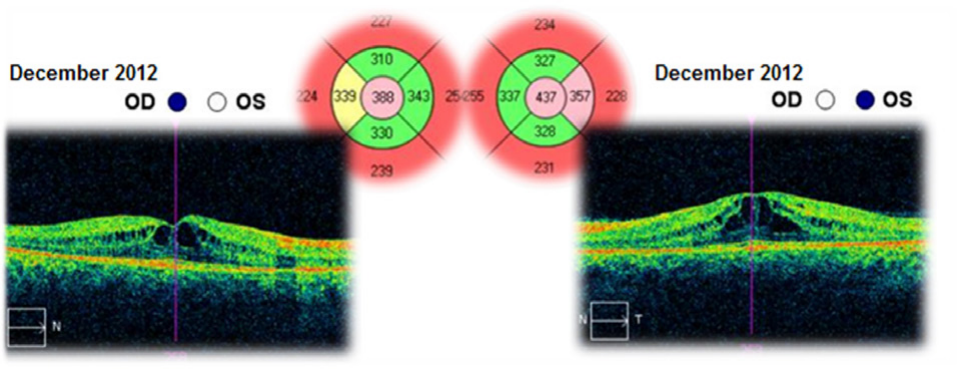
Macular OCT in December 2012 – Cystoid macular oedema – Central retinal thickness is lower than in the previous scan (October 2012) – with 86μm in the right eye and 64μm in the left eye

**Fig. 12:**
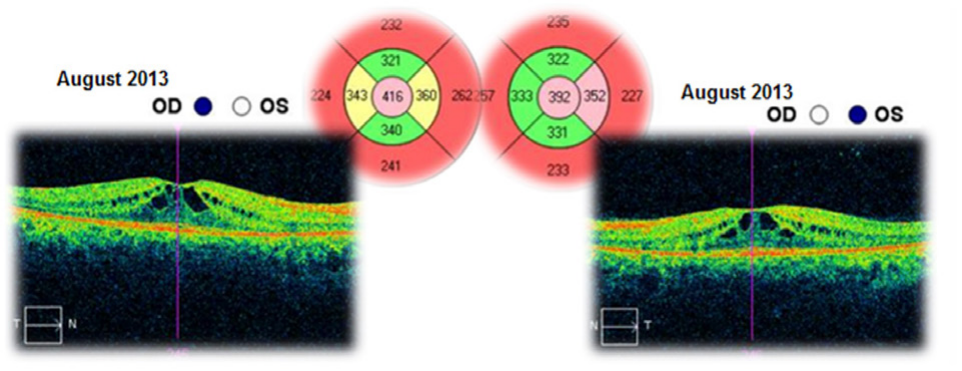
Macular OCT in August 2013 – Cystoid macular oedema – Compared to the previous scan (December 2012), the central retinal thickness is higher with 28μm in the right eye and lower with 45μm in the left eye

**Fig. 13:**
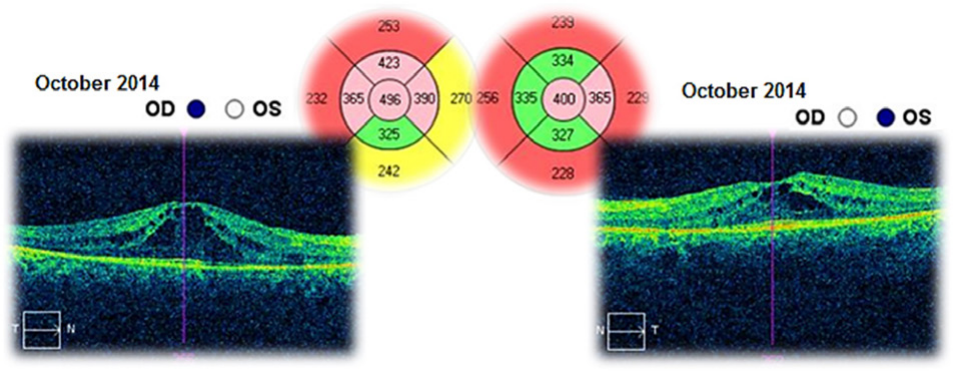
Macular OCT in October 2014 – Cystoid macular oedema – Compared to the previous scan (August 2013), the central retinal thickness is higher with 80μm in right eye and it is almost unchanged in the left eye

**Fig. 14:**
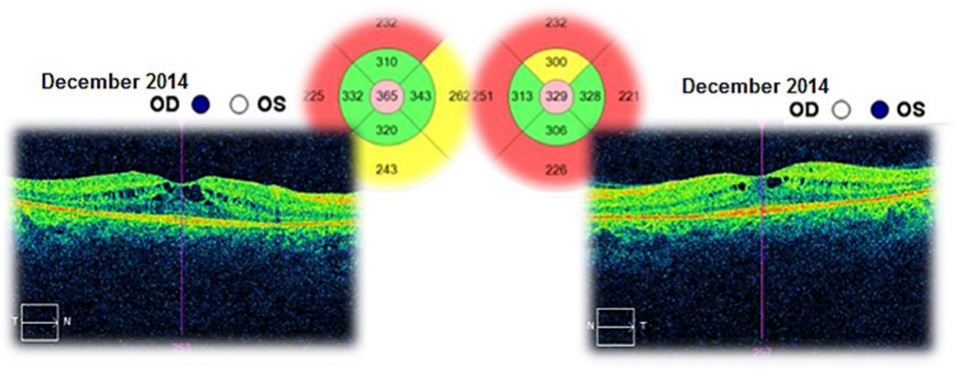
Macular OCT in December 2014 – Cystoid macular oedema – Central retinal thickness is lower than in the previous scan (October 2014), with 131μm in the right eye and with 71μm in the left eye

**Fig. 15:**
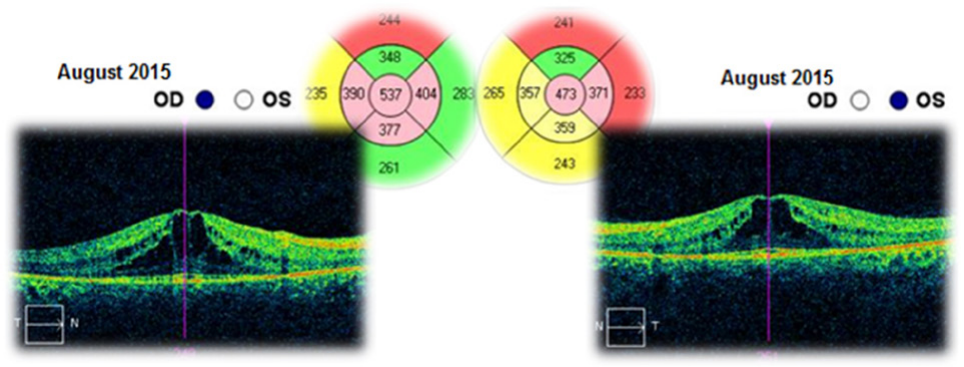
Macular OCT in August 2015 – Cystoid macular oedema – Central retinal thickness is higher than in the previous scan (December 2014), with 172μm in the right eye and with 144μm in the left eye

## Discussion

The diagnosis of Retinitis Punctata Albescens is rather more difficult than in classical cases of Retinitis Pigmentosa pathognomonic pigmentary fundus changes. The association with the optic disc drusen is not uncommon [**5**-**8**].

Cystoid macular oedema has been reported to be associated with Retinitis Pigmentosa, the prevalence of unilateral macular cysts may be up to 38% and 27% in bilateral involvement [**9**]. Despite the intraretinal cysts and fluid accumulation in the macula, visual acuity is preserved (20/ 20 at presentation in both eyes and between 20/ 20 and 20/ 25 in the 3 years of follow-up period). This is due to the sparing of the foveal zone from the photoreceptor loss [**9**].

As a particularity in this case, the late onset of symptomatology was also noticed in the 4th decade but with severe structural impairment and important peripheral visual field loss.

As the patient was only 37 years old, the long-time visual prognosis was reserved. Visual loss was correlated with the progression of retinal degeneration to the macula, loss of foveal photoreceptors, evolution of cystoid macular oedema and appearance of posterior subcapsular cataracts [**10**].

Further molecular genetic examinations are required to be performed in order to establish the gene mutation and to refer the patient for genetic counseling.

## Financial Disclosures

None of the other authors has any financial or proprietary interests to disclose.
